# A united statement of the global chiropractic research community against the pseudoscientific claim that chiropractic care boosts immunity

**DOI:** 10.1186/s12998-020-00312-x

**Published:** 2020-05-04

**Authors:** Pierre Côté, André Bussières, J. David Cassidy, Jan Hartvigsen, Greg N. Kawchuk, Charlotte Leboeuf-Yde, Silvano Mior, Michael Schneider, and more than 140 signatories# call for an end to pseudoscientific claims on the effect of chiropractic care on immune function, Luc Aillet, Carlo Ammendolia, Bodil Arnbak, Iben Axen, Mirjam Baechler, Florian Barbier-Cazorla, Gaëtan Barbier, Cecilia Bergstrøm, Amber Beynon, Marc-André Blanchette, Philip S. Bolton, Alan Breen, Johanne Brinch, Gert Bronfort, Benjamin Brown, Paul Bruno, Mikkel Brunsgaard Konner, Christopher Burrell, Jason W. Busse, David Byfield, Marco Campello, Carol Cancelliere, Linda Carroll, Christine Cedraschi, Charlène Chéron, Ngai Chow, Henrik Wulff Christensen, Stine Claussen, Melissa Corso, Matthew A. Davis, Marine Demortier, Diana De Carvalho, Katie de Luca, Annemarie de Zoete, Klaus Doktor, Aron Downie, Alister du Rose, Andreas Eklund, Roger Engel, Mark Erwin, James E. Eubanks, Roni Evans, Will Evans, Matthew Fernandez, Jonathan Field, Gilles Fournier, Simon French, Signe Fuglkjaer, Olivier Gagey, Rosemary Giuriato, Jordan A. Gliedt, Christine Goertz, Guillaume Goncalves, Diane Grondin, Mark Gurden, Mitchell Haas, Scott Haldeman, Steen Harsted, Lisbeth Hartvigsen, Jill Hayden, Cesar Hincapié, Jeffrey J. Hébert, Bue Hesby, Lise Hestbæk, Sheilah Hogg-Johnson, Maria A. Hondras, Margaux Honoré, Samuel Howarth, H. Stephen Injeyan, Stanley Innes, Pernille Marie Irgens, Craig Jacobs, Hazel Jenkins, Alan Jenks, Tue Secher Jensen, Melker Johhansson, Alice Kongsted, Deborah Kopansky-Giles, Rikke Kryger, Arnaud Lardon, Henrik Hein Lauridsen, Brent Leininger, Nadège Lemeunier, Christine Le Scanff, Eugene A. Lewis, Kathleen Linaker, Lise Lothe, Andrée-Anne Marchand, David McNaughton, Anne-Laure Meyer, Peter Miller, Anne Mølgaard, Craig Moore, Donald R. Murphy, Corrie Myburgh, Birgitte Myhrvold, Dave Newell, Genevieve Newton, Casper Nim, Margareta Nordin, Luana Nyiro, Søren O’Neill, Cecilie Øverås, Isabelle Pagé, Mégane Pasquier, Charles W. Penza, Stephen M. Perle, Mathieu Picchiottino, Mathieu Piché, Erik Poulsen, Jeffrey Quon, Tim Raven, Mana Rezai, Eric J. Roseen, Sidney Rubinstein, Louis-Rachid Salmi, Petra Schweinhardt, Heather M. Shearer, Laura Sirucek, Delphine Sorondo, Paula J. Stern, Joel Stevans, Mette Jensen Stochkendahl, Kent Stuber, Maja Stupar, John Srbely, Michael Swain, Julita Teodorczyk-Injeyan, Jean Théroux, Haymo Thiel, Lars Uhrenholt, Anneke Verbeek, Leslie Verville, Karl Vincent, Andrew L. Dan Wang, Kenneth A. Weber, James M. Whedon, Jessica Wong, Francesca Wuytack, James Young, Hainan Yu, Dorte Ziegler

**Affiliations:** 1 Faculty of Health Sciences, Ontario Tech University, Oshawa, Canada; 2 Centre for Disability Prevention and Rehabilitation at Ontario Tech University and CMCC, Oshawa, Canada; 3 Division of Epidemiology, Dalla Lana School of Public Health, University of Toronto, Toronto, Canada; 4 Département chiropratique, Université du Québec à Trois-Rivières, Trois-Rivières, Canada; 5 School of Physical and Occupational Therapy, Faculty of Medicine McGill University, Montreal, Canada; 6 Department of Sports Science and Clinical Biomechanics, University of Southern Denmark, Odense, Denmark; 7 Nordic Institute of Chiropractic and Clinical Biomechanics, Odense, Denmark; 8 Faculty of Rehabilitation Medicine, University of Alberta, Edmonton, Canada; 9 Institute for Regional Health Research, University of Southern Denmark, Odense, Denmark; 10 Canadian Memorial Chiropractic College, Toronto, Canada; 11 School of Health and Rehabilitation Sciences, University of Pittsburgh, Pittsburgh, USA; 12 Clinical and Translational Science Institute, University of Pittsburgh, Pittsburgh, USA

**Keywords:** Chiropractic, Spinal manipulation, Immunity, Pseudoscience, Coronavirus

## Abstract

**Background:**

In the midst of the coronavirus pandemic, the International Chiropractors Association (ICA) posted reports claiming that chiropractic care can impact the immune system. These claims clash with recommendations from the World Health Organization and World Federation of Chiropractic. We discuss the scientific validity of the claims made in these ICA reports.

**Main body:**

We reviewed the two reports posted by the ICA on their website on March 20 and March 28, 2020. We explored the method used to develop the claim that chiropractic adjustments impact the immune system and discuss the scientific merit of that claim. We provide a response to the ICA reports and explain why this claim lacks scientific credibility and is dangerous to the public. More than 150 researchers from 11 countries reviewed and endorsed our response.

**Conclusion:**

In their reports, the ICA provided no valid clinical scientific evidence that chiropractic care can impact the immune system. We call on regulatory authorities and professional leaders to take robust political and regulatory action against those claiming that chiropractic adjustments have a clinical impact on the immune system.

## Background

We are currently facing the greatest global public health crisis in a century. Fighting the coronavirus pandemic has required that we change the way we live and observe strict public health guidelines. This is necessary because, at this time, there are no effective vaccines, treatments or cures for COVID-19 [1, 2]. Chiropractors, as members of the health care system, should disseminate the best available public health information to the public [3]. Any attempt to behave otherwise can be misleading and potentially dangerous to individual patients and the public at large.

On March 20, 2020, the International Chiropractors Association (ICA), a US based chiropractic organization, posted a report claiming that chiropractic adjustments can boost immune function with the implication that it might be helpful in preventing COVID-19 [4]. In their report, the ICA states that: “*Although there are no clinical trials to substantiate a direct causal relationship between the chiropractic adjustment and increased protection from the COVID-19 virus, there is a growing body of evidence that there is a relationship between the nervous system and the immune system*” and “*The observation that those who use chiropractic regularly and do not become ill with cold, flu, or other community shared illnesses is frequent within the profession and should not be ignored”* [4]. The ICA position directly contradicts the World Health Organization (WHO) that unequivocally states that “*there are no effective health interventions to prevent or treat coronavirus infections*” [1, 2], and the World Federation of Chiropractic (WFC) that states that “*there is no credible scientific evidence that chiropractic spinal adjustments/manipulations confers or boosts immunity”* [3].

On March 28, 2020, the ICA posted a revised report which reiterated the information included in the first report, with the addition of references supporting the link between chiropractic care and immune function [5]. In both reports, the ICA claims that their review of the literature confirms “*An association between spinal manipulation and the autonomic nervous system” and that “These studies suggest mechanisms by which spinal influences may mediate a clinically significant impact on immune function.*” Therefore, the main message of both reports is that chiropractic care can have a clinically meaningful impact on immune system function. We discuss the scientific validity of the claims made by the ICA.

## Main body

We investigated the approach used by the ICA to support their claim that chiropractic adjustments impact the immune system. We compared the ICA claim to the findings and conclusions of one systematic review of the literature on the effect of spinal manual therapies on autonomic nervous system activity [6] and two systematic reviews on the efficacy and effectiveness of chiropractic treatment and manual therapy on the prevention and treatment of non-musculoskeletal disorders [7, 8]. Further, we used a list of warning signs of pseudoscience to assess the scientific merit of the claims [9]. Finally, 153 researchers from 11 countries (8 co-authors and 145 signatories) who are involved in research relevant to chiropractic reviewed and endorsed our response.

While the ICA states *“that no claims can be made about COVID-19 and chiropractic*”*,* their report implies that chiropractic adjustments can boost the immune system through its effect on the nervous system. The ICA claim rests on two assumptions: i) chiropractic adjustments have a beneficial effect on the nervous system and ii) chiropractic adjustments will improve the immune system through the nervous system. These assumptions are not supported by robust evidence that chiropractic adjustments are efficacious or effective in improving immune function [6–8]. We consider that proclaiming the benefits of chiropractic adjustment/spinal manipulation on immunity during a pandemic is plainly irresponsible and demonstrates a lack of understanding of science, the coronavirus pandemic and public health risks.

Our critical review of the reports suggest that the ICA created a positive narrative for the effect of chiropractic adjustments and immune function report by selectively assembling a series of unconnected basic science studies [4, 5]. This strategy, called “emphasis on confirmation”, is a warning sign of pseudoscience [9]. Moreover, this approach fails to respect the established boundaries that exist between basic and clinical research. For example, two of the basic science studies included in the ICA report were led by one of the signatories of this commentary, Stephen Injeyan DC, PhD [10, 11]. According to Dr. Injeyan: *“No published studies have so far demonstrated the clinical significance of spinal manipulation and immune enhancement, our research included. Our studies were conducted in asymptomatic subjects, in vitro cellular models, and the outcomes were measured shortly following SMT. There are no parallels between our experimental research and clinical care.”* By only citing basic science experiments, the ICA appear to have overlooked the WHO guidance on implementation research, which clearly states that basic science experiments do not provide relevant justification for implementation of a health intervention [12].

Any health care intervention must be evaluated for its clinical efficacy and effectiveness in well-designed randomized controlled trials before it is implemented in clinical practice [12]. This requirement is not new; it was first implemented by the US Food and Drug Administration in 1962 [13]. With this in mind, it is all the more noteworthy that none of the studies cited in the ICA report provide evidence that chiropractic adjustments actually prevent the onset of infectious diseases in healthy individuals, or improve the health of patients suffering from a viral infection. We call on the ICA to explain why it does not adhere to internationally accepted standards of research implementation but instead rely on unconnected basic science studies when linking chiropractic care to immune system function.

The ICA also relied on anecdotal evidence to support their claim; this is another warning sign of pseudoscience [9]. For example, the authors state: *“The observation that those who use chiropractic regularly and do not become ill with colds, flu, and other community shared illnesses is frequent within the profession and should not be ignored”* [4, 5]. At best, this type of anecdotal evidence is useful to generate research hypotheses to be tested in high quality randomized clinical trials. To our knowledge, the hypothesis that chiropractic care reduces the risk of becoming ill with viral colds, flu, and other community shared illnesses has never been properly tested. Any claims suggesting otherwise lack scientific merit and should not be used to justify treating patients with chiropractic adjustments.

Advancing extraordinary claims without providing extraordinary evidence should raise significant concerns about the scientific validity of the ICA’s position. In their reports, the ICA claims that individuals who received chiropractic care during the 1918 Spanish flu pandemic were 51 to 91 times less likely to die than those who were treated by medical doctors [4, 5]. These effect sizes are too large to be trustworthy and are a red flag of pseudoscience, because extraordinary claims require extraordinary evidence [9]. Using data from a 100-year-old non-published, non-randomized controlled trial to suggest that chiropractic adjustments reduces mortality from the flu is scientifically and socially irresponsible.

Pseudoscience has the potential to mislead and misinform at any time; even more so in the midst of a pandemic when the public is vulnerable. The current coronavirus pandemic demands that we act responsibly by adopting sound public health practices as recommended by the WHO [14]. These include but are not restricted to regular handwashing, respiratory etiquette, physical distancing, staying at home, limiting trips outside the home except to obtain food or medicine and wearing a mask if symptomatic [14]. We have seen widespread adherence to the guidance around COVID-19, but as scientists and clinicians we have a public health duty to sound the alarm and denounce pseudoscientific claims such as the ones made by the ICA in its reports.

## Conclusion

We call on regulatory authorities and professional leaders to take appropriate political and regulatory action against those making direct or indirect unsubstantiated claims that spinal adjustments can boost immunity, or benefit patients with infectious diseases, especially coronavirus infections. Above all, these actions must aim to protect the safety and well-being of patients and the public.

## Data Availability

Not applicable.

## Abbreviations

FDA: Food and Drug Administration
ICA: International Chiropractors Association
WHO: World Health Organization
WFC: World Federation of Chiropractic

## References

[1] World Health Organization (2020). Q&A on coronaviruses (COVID-19).

[2] World Health Organization (2020). Coronavirus disease (COVID-19) advice for the public: Myth busters.

[3] World Federation of Chiropractic (2020). Coronavirus Disease 2019 (COVID-19). Advice for chiropractors.

[4] International Chiropractors Association (2020). Immune function and chiropractic what does the evidence provide?.

[5] International Chiropractors Association (2020). Immune function and chiropractic what does the evidence provide?.

[6] Picchiottino M, Leboeuf-Yde C, Gagey O, Hallman DM (2019). The acute effects of joint manipulative techniques on markers of autonomic nervous system activity: a systematic review and meta-analysis of randomized sham-controlled trials. Chiropr Man Therap.

[7] Goncalves G, Le Scanff C, Leboeuf-Yde C (2018). Effect of chiropractic treatment on primary or early secondary prevention: a systematic review with a pedagogic approach. Chiropr Man Therap.

[8] Clar C, Tsertsvadze A, l Court R, Hundt GL, Clarke A, Sutcliffe P (2014). Clinical effectiveness of manual therapy for the management of musculoskeletal and non-musculoskeletal conditions: systematic review and update of UK evidence report. Chiropr Man Therap.

[9] Lilienfeld SO, Ammirati R, David M (2012). Distinguishing science from pseudoscience in school psychology: science and scientific thinking as safeguards against human error. J Sch Psychol.

[10] Teodorczyk-Injeyan JA (2018). Elevated production of nociceptive CC chemokines and sE-selectin in patients with low back pain and the effects of spinal manipulation: a nonrandomized clinical trial. Clin J Pain.

[11] Teodorczyk-Injeyan JA, Injeyan HS, Ruegg R (2006). Spinal manipulative therapy reduces inflammatory cytokines but not substance P production in normal subjects. J Manip Physiol Ther.

[12] Peters DH, Tran NT, Adam T (2013). Implementation research in health: a practical guide. Alliance for Health Policy and Systems Research, World Health Organization.

[13] Junod W (2020). FDA and clinical drug trials: a short history.

[14] World Health Organization, 2020 (2020). Coronavirus disease (COVID-19) technical guidance: patient management.

